# Monoamine Levels and Parkinson’s Disease Progression: Evidence From a High-Performance Liquid Chromatography Study

**DOI:** 10.3389/fnins.2021.605887

**Published:** 2021-07-29

**Authors:** Patsorn Wichit, Sekh Thanprasertsuk, Onanong Phokaewvarangkul, Roongroj Bhidayasiri, Saknan Bongsebandhu-phubhakdi

**Affiliations:** ^1^Department of Physiology, Faculty of Medicine, Chulalongkorn University, Bangkok, Thailand; ^2^Faculty of Physical Therapy, Huachiew Chalermprakiet University, Bang Phli, Thailand; ^3^Chulalongkorn Cognitive Clinical and Computational Neuroscience Special Task Force Research Group, Chulalongkorn University, Bangkok, Thailand; ^4^Chula Neuroscience Center, King Chulalongkorn Memorial Hospital, The Thai Red Cross Society, Bangkok, Thailand; ^5^Chulalongkorn Centre of Excellence for Parkinson’s Disease & Related Disorders, King Chulalongkorn Memorial Hospital, The Thai Red Cross Society, Bangkok, Thailand; ^6^Division of Neurology, Department of Medicine, Faculty of Medicine, Chulalongkorn University, Bangkok, Thailand

**Keywords:** Parkinson’s disease, monoamine, dopamine, norepinephrine, epinephrine, serotonin

## Abstract

Parkinson’s disease (PD) is associated with dysfunction of monoamine neurotransmitter systems. We investigated changes in the levels of monoamine and their metabolites in PD patients, together with their association to clinical profiles. PD patients and age-matched control subjects (*n* = 40 per group) were enrolled. Using high-performance liquid chromatography (HPLC) with an electrochemical detector, levels of monoamines (dopamine, DA; norepinephrine, NE; epinephrine, EPI; and serotonin, 5-HT) were measured in plasma, while the metabolites (homovanillic acid, HVA; vanillylmandelic acid, VMA; and 5-hydroxyindoleacetic acid, 5-HIAA) were measured in urine. Plasma DA level was not significantly different between PD and control groups. PD patients had significantly higher plasma NE but lower EPI and 5-HT levels. PD patients had a significantly higher HVA/DA ratio and lower VMA/NE ratio than control subjects, while the 5-HIAA/5-HT ratio was not different between the groups. Regarding the association between monoamine levels and clinical profiles, the DA level had a negative relationship with disease duration and the 5-HT level had a negative relationship with severity of motor impairment. These findings emphasized the involvements of several neurotransmission systems and their association with clinical profiles in PD patients, demonstrated by quantification of monoamine levels in peripheral body fluids. This could benefit appropriate pharmacological treatment planning in respect of monoamine changes and might also help predict subsequent clinical symptoms.

## Introduction

Parkinson’s disease (PD) is a neurodegenerative disorder characterized by motor impairments including bradykinesia, rigidity, rest tremor, and postural instability, combined with a variety of non-motor symptoms (Przedborski, 2017). Hallmark pathologies of PD are Lewy body deposition and progressive deterioration of dopaminergic neurons in substantia nigra, leading to depletion of central dopamine (DA) level (Braak et al., 2003). There is no specific test for PD diagnosis nowadays. Neurologists usually diagnose PD based on clinical assessment and dopaminergic medication responsiveness.

Apart from abnormality of DA, neuroimaging and post-mortem brain tissue studies have demonstrated imbalances of other monoamine neurotransmitters in PD including norepinephrine (NE), epinephrine (EPI), and serotonin (5-HT) (Barone, 2010). Thus, management of PD has focused on several affected neurotransmitter systems rather than the dopaminergic system alone (Barone, 2010). However, there is lack of information on the alteration of monoamine levels measuring in peripheral body fluids in different stages of PD. In addition, many reports showed contradictory information regarding the association between monoamine dysfunctions and clinical profiles of PD such as age, gender, medications, disease duration, and severity (Lunardi et al., 2009; Olivola et al., 2014; Kaasinen and Vahlberg, 2017).

The objective of this study was to investigate the alteration of DA, NE, EPI, and 5-HT levels in plasma along with levels of their metabolites in the urine of PD patients. Additionally, we aimed to compare the levels of monoamines in early and advanced stages and also evaluate their associations with clinical profiles including age, gender, L-DOPA equivalent daily dose (LEDD), disease duration, and motor severity.

## Materials and Methods

### Participants

Participants in this study were male and female PD patients aged between 30 and 80 years and healthy gender- and age-matched control groups. Based on a previous report (Tong et al., 2015), the sample size estimation for our primary objective (PD versus control groups) was 16 participants per group (α = 0.05, 80% power). However, our secondary objective was to compare the monoamine levels between early- and advanced-stage patients in the PD group. We thus recruited the PD patients until there were at least 16 participants categorized in either early or advanced stages. Eventually, there were 40 participants in the PD group (24 and 16 participants categorized in early and in advanced stages, respectively) and 40 participants in the control group. Patients were diagnosed with PD according to the UK Parkinson’s Disease Society Brain Bank Clinical Diagnostic Criteria and recruited between February 2018 and November 2019 from the outpatient clinic at Chulalongkorn Centre of Excellence on Parkinson’s Disease and Related Disorders, King Chulalongkorn Memorial Hospital, Thailand.^1^ Demographic information and clinical data including disease duration, comorbidities, current medications, and LEDD were recorded. Disease severity was assessed based on the modified Hoehn and Yahr (HY) scale and was classified as early and advanced stages when scales were 1–2.5 and 3–5, respectively. Cognitive function was evaluated using the Thai Version of Mini-Mental State Examination (MMSE-Thai 2002). Patients were excluded when (1) they had other identified central nervous system abnormalities such as cognitive disorders, cerebrovascular disease, and traumatic brain injury; (2) they had psychiatric comorbidities; and (3) they were taking medications which possibly interfere the monoamine concentration including selective 5-HT reuptake inhibitors, 5-HT-NE reuptake inhibitors, tricyclic antidepressants, neuroleptics, and β-adrenergic antagonists. Participants had discontinued parkinsonian drugs or other medications which could disturb monoamine levels at least 12 h prior to collection of the specimens, as stated in a previous study (Lian et al., 2018). Foods and beverages including coffee, tea, banana, chocolate, vanilla, and citrus fruits were also restricted during this period.

### Neurotransmitter and Metabolite Levels Determination

#### Specimen Collection

Plasma and urine were obtained to determine the neurotransmitter and metabolite concentrations, respectively. Blood samples (3 ml) were collected from a cubital vein, drawn into an EDTA tube, immediately centrifuged to separate plasma at 3,000 × *g* (4°C) for 10 min, and stored at −80°C until analyzed. Plasma DA, NE, EPI, and 5-HT were measured by HPLC with an electrochemical detector. At the same time, a single urine sample was collected in a container with 10 ml (32%) hydrochloric acid per liter of urine and pH was adjusted to 1–2. In these urine samples, the levels of homovanillic acid (HVA), vanillylmandelic acid (VMA), and 5-hydroxyindoleacetic acid (5-HIAA), metabolites of DA, NE/EPI, and 5-HT, respectively, were quantified.

#### HPLC Analysis

Levels of the neurotransmitter and metabolite were determined by the *ClinRep*^®^ Complete Kits for catecholamines, 5-HT, and VMA/HVA/5-HIAA (*München, Germany*). Analysis was achieved with HPLC *Chrome* systems (*Gräfelfing, Germany*), consisting of isocratic pump CLC 300, programmable autosampler injection CLC 200, and electrochemical detector model CLC 100. The chromatographic peaks were separated by the *Recipe*’s special reversed-phase columns (*München, Germany*) and peaks identified by the *Easyline* analysis software program (*München, Germany*). Using an internal standard technique, monoamine concentrations were calculated by comparing the retention time and peak area with a calibration curve. Sample preparations and monoamine concentration measurements were performed according to the manufacturer’s instructions as previously described (Andersen et al., 2017). Briefly, for determination of DA, NE, and EPI, plasma (1 ml) and an internal standard (50 μl) were centrifuged with a washing solution and eluting reagent. The eluted sample (40 μl) was then injected to an electrochemical detector (500 mV, 1 nA) at a flow rate of 1 ml/min. For analysis of 5-HT, plasma (200 μl) was mixed and centrifuged with internal standard (10 μl) and precipitant solution (200 μl). Supernatant (20 μl) was injected at a flow rate of 1 ml/min to the electrochemical detector (450 mV, 20 nA). For HVA/VMA/5-HIAA analyses, the urine sample (50 ml) and internal standard (1 ml) were mixed, washed, and added with the eluting reagent. The eluted solution (20 μl) was then injected to the electrochemical detector (800 mV, 50 nA) at a flow rate of 0.9 ml/min.

### Statistical Analysis

We performed statistical analysis with *SPSS Statistics 22* (*IBM Corporation, New York, NY, United States*). All data were tested for normal distribution using Kolmogorov–Smirnov. The demographic data, monoamine levels, metabolite levels, and ratios between PD and control groups were compared using the independent *t*-test (two-tailed) for parametric data or Mann–Whitney *U*-test for non-parametric data. Comparisons of plasma monoamine levels between early and advanced stages of PD patients were also performed by the Mann–Whitney *U*-test. The relationships between plasma monoamine levels and clinical profiles were tested by using multiple linear regression models with the stepwise method. Plasma monoamine levels were log-transformed to fit the linear model. Age, gender, LEDD, disease duration, and motor severity were set as the covariates. The standardized coefficients (β), 95% confidence interval (CI), and coefficient of multiple determination (*R*^2^) were presented. Statistical significance was defined as *p*-value < 0.05.

## Results

### Demographic Data and Clinical Profiles

The mean ages of control subjects and PD patients were 55.5 ± 6.3 and 57.6 ± 8.5 years, respectively (*p* = 0.22). The majority of participants were male in both groups. In the PD group, the average disease duration was 13.2 ± 7.1 years and the mean LEDD was 1055.3 ± 656.9 mg/day (Table 1). Other PD-related medications including trihexyphenidyl 1–2 mg/day and clonazepam 0.25–2 mg/day had been taken by 6 (15.0%) and 15 (37.5%), respectively. In the PD group, histories of essential hypertension, type 2 diabetes mellitus, and hypercholesterolemia were documented in four (10.0%), two (5.0%), and three (7.5%) patients, respectively. Medications taken for their underlying diseases were amlodipine 5–10 mg/day in three (7.5%), enalapril 10 mg/day in one (2.5%), losartan 50 mg/day in one (2.5%), metformin 500–1,000 mg/day in two (5.0%), and statins in three (7.5%) patients.

**TABLE 1:** Demographic data and clinical characteristics of control and PD groups.

Characteristics		Control (*n* = 40)	Parkinson (*n* = 40)	*p*-value
Age [years, mean ± SD]		55.50 ± 6.33	57.55 ± 8.48	0.22^a^
Males: females		22:18	27:13	0.30^b^
MMSE score [mean ± SD]		28.08 ± 1.94	28.50 ± 1.52	0.46^a^
Disease duration [years, mean ± SD]		NA	13.18 ± 7.11	–
**Modified H&Y stages of PD**				
- Average modified H&Y stage [mean ± SD]		NA	2.76 ± 1.06	–
- Frequency of patients [N (%)]				
	Stage 1	NA	3 (7.5%)	–
	Stage 1.5	NA	2 (5%)	–
	Stage 2	NA	6 (15%)	–
	Stage 2.5	NA	13 (32%)	–
	Stage 3	NA	8 (20%)	–
	Stage 4	NA	4 (10%)	–
	Stage 5	NA	4 (10%)	–
LEDD [mg/day, mean ± SD]		NA	1055.3 ± 656.9	–

*MMSE = Mini-Mental State Examination; LEDD = L-DOPA-equivalent daily dose; SD = standard deviation.*

*^a^Independent t-test.*

*^b^Chi-square test.*

PD patients were classified into two disease severity subgroups; 24 (60%) patients were in the early stage (modified HY stages 1–2.5), and 16 (40%) patients were in the advanced stage (modified HY stages 3–5). Early-stage patients were younger, had a shorter disease duration, and had a lower LEDD than the advanced-stage ones (Table 2). There were no significant differences in the proportion of patients taking trihexyphenidyl (20.8 and 6.2%, *p* = 0.37) and clonazepam (37.5 and 37.5%, *p* = 1.00) between the early and advanced subgroups, respectively.

**TABLE 2:** Demographic and clinical characteristics of early and advanced stage PD patients.

Characteristics	Early stage (*n* = 24)	Advanced stage (*n* = 16)	*p*-value
Age [years, mean ± SD]	54.3 ± 8.5	62.5 ± 5.7	0.001**^a^
Males: females	18:6	9:7	0.30^b^
Disease duration [years, mean ± SD]	10.8 ± 5.7	16.8 ± 7.7	0.01*^a^
LEDD [mg/day, mean ± SD]	886.6 ± 472.1	1,338.3 ± 798.3	0.04*^a^

*LEDD = L-DOPA-equivalent daily dose; SD = standard deviation.*

*^a^Independent t-test.*

^b^Chi-square test.

*p<0.05; **p<0.01.

### Comparisons of Plasma Monoamine Levels Between PD and Control Groups

**FIGURE 1:**
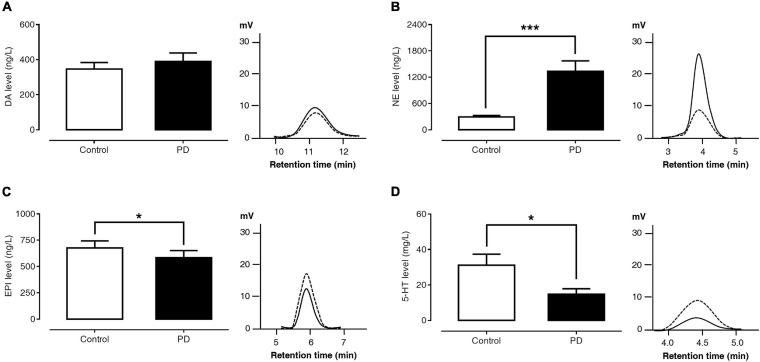
Comparisons of plasma DA **(A)**, NE **(B)**, EPI **(C)**, and 5-HT **(D)** levels and HPLC chromatograms between control subjects (dash lines) and PD patients (solid lines). Data are presented as mean ± SEM (*p < 0.05, ***p < 0.001).

Figures 1A–D show the levels of plasma monoamine and represent their HPLC chromatograms of the control and PD groups. The plasma DA level was not significantly different between PD patients and control subjects (389.85 ± 48.06 versus 346.45 ± 37.37 ng/l, *p* = 0.864). The plasma NE level was significantly higher in PD patients than in control subjects (1,336.72 ± 235.87 versus 295.48 ± 31.14 ng/l, *p* < 0.001). Compared to control subjects, PD patients had a significantly lower plasma EPI (584.70 ± 66.84 versus 676.73 ± 66.81 ng/l, *p* = 0.027) and 5-HT levels (14.81 ± 3.11 versus 31.20 ± 6.15 μg/l, *p* = 0.014).

### Comparisons of Urinary Metabolite Levels Between PD and Control Groups

**FIGURE 2:**
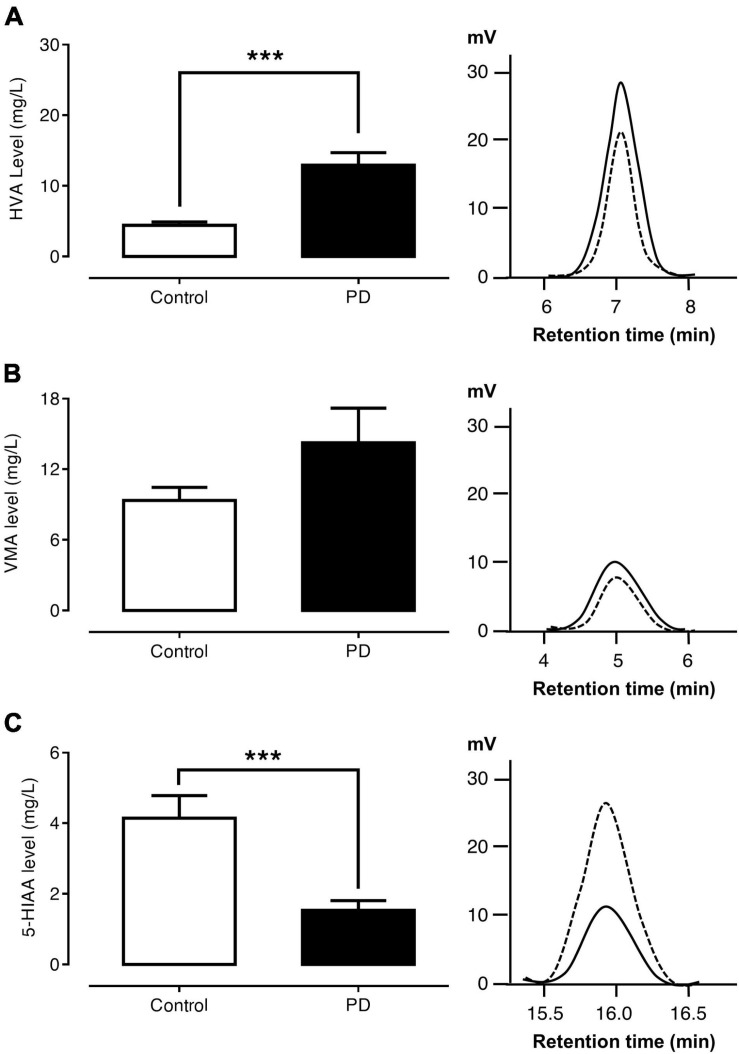
Comparisons of urinary HVA **(A)**, VMA **(B)**, and 5-HIAA **(C)** levels and HPLC chromatograms between control subjects (dash lines) and PD patients (solid lines). Data are presented as mean ± SEM (***p < 0.001).

Figures 2A–C show the levels of urinary HVA, VMA, and 5-HIAA and the HPLC chromatograms of the control and PD groups, respectively. The urinary HVA level was significantly higher in PD patients than in control subjects (12.94 ± 1.78 versus 4.43 ± 0.45 mg/l, *p* < 0.001). The urinary VMA level was not significantly different between the PD and control groups (14.26 ± 2.94 versus 9.36 ± 1.10 mg/l, *p* = 0.917). On the other hand, the urinary 5-HIAA level was significantly lower in PD patients than in control subjects (1.54 ± 0.27 versus 4.14 ± 0.63 mg/l, *p* < 0.001).

### Comparisons of the Metabolite/Monoamine Ratio Between PD and Control Groups

**FIGURE 3:**
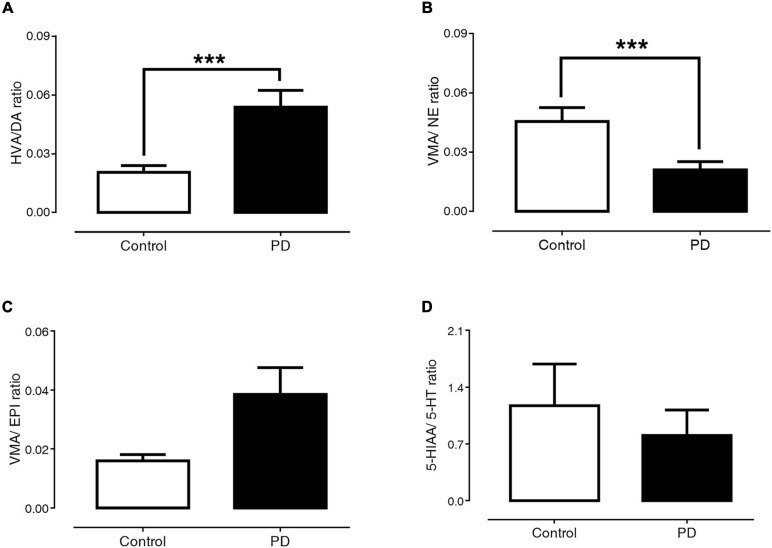
Comparisons of HVA/DA **(A)**, VMA/NE **(B)**, VMA/EPI **(C)**, and 5-HIAA/5-HT **(D)** ratio between control subjects and PD patients. Data are presented as mean ± SEM (***p < 0.001).

Figures 3A–D exhibit the ratio of HVA/DA, VMA/NE, VMA/EPI, and 5-HIAA/5-HT, respectively. The findings showed that PD patients had a significantly higher HVA/DA ratio than control subjects (0.054 ± 0.009 versus 0.021 ± 0.003, *p* < 0.001). In contrast, the VMA/NE ratio of PD patients was significantly lower than that of control subjects (0.021 ± 0.004 versus 0.045 ± 0.007, *p* < 0.001). The ratios of VMA/EPI (0.039 ± 0.009 versus 0.016 ± 0.002, *p* = 0.29) and 5-HIAA/5-HT (0.804 ± 0.315 versus 1.171 ± 0.514, *p* = 0.74) were not significantly different between the PD and control groups.

### Association Between Plasma Monoamine Levels and Clinical Profiles of PD Patients

**FIGURE 4:**
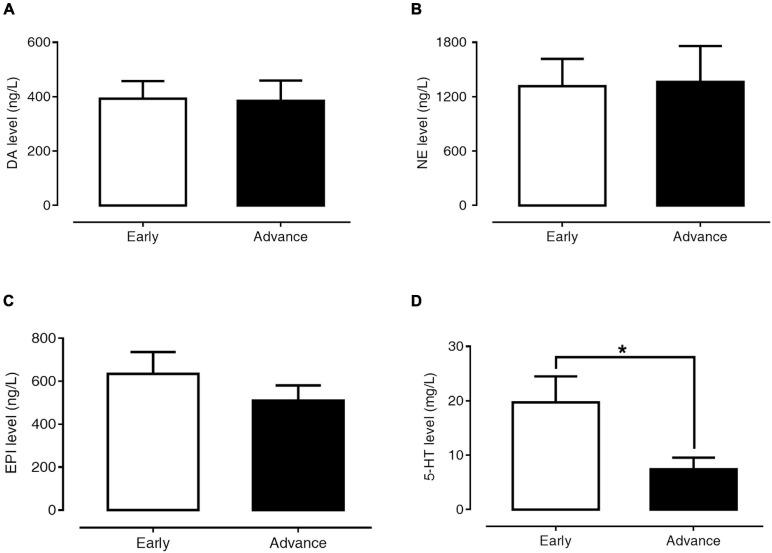
Levels of plasma DA **(A)**, NE **(B)**, EPI **(C)**, and 5-HT **(D)** in early- and advanced-stage PD patients. Data are presented as mean ± SEM (*p < 0.05).

Figures 4A–D present the levels of plasma DA, NE, EPI, and 5-HT in early-stage and advanced-stage PD patients. Between these two subgroups, there were no significant differences in plasma levels of DA (393.12 ± 64.12 versus 384.94 ± 74.52 ng/l, *p* = 0.984), NE (1,317.84 ± 298.83 versus 1,365.05 ± 395.14 ng/l, *p* = 0.624), and EPI (634.66 ± 100.75 versus 509.77 ± 71.18 ng/l, *p* = 0.935). However, plasma 5-HT levels in advanced PD patients were significantly lower than in early-stage patients (7.44 ± 2.11 versus 19.72 ± 4.77 μg/l, *p* = 0.024).

The contributions of clinical profiles, including age, gender, LEDD, disease duration, and motor severity, to plasma monoamine levels were evaluated by multiple linear regression analyses (Table 3). From these analyses, we found that disease duration had a negative relationship with plasma DA level (β = −0.328, 95% CI [−0.033, −0.002], *p* = 0.025) and motor severity had a negative relationship with plasma 5-HT level (β = −0.351, 95% CI [−0.910, −0.060], *p* = 0.026). LEDD was also the significant determinant of both plasma DA (β = 0.379, 95% CI [0.118, 0.835], *p* = 0.011) and NE levels (β = 0.394, 95% CI [0.141, 1.059], *p* = 0.012).

**TABLE 3:** Multiple linear regression model for the association between plasma monoamine levels and clinical profiles of PD patients.

Dependent variables	Independent variables	Standardized coefficients (β)	95% CI	*p*-value
Plasma DA level (*R*^2^ = 0.274)	LEDD	0.379	0.118, 0.835	0.011*
	Disease duration	−0.328	−0.033, −0.002	0.025*
Plasma NE level (*R*^2^ = 0.155)	LEDD	0.394	0.141, 1.059	0.012*
Plasma 5-HT level (*R*^2^ = 0.123)	Motor severity (0 = early, 1 = advanced)	−0.351	−0.910, −0.060	0.026*

CI = confidence interval; R^2^ = coefficient of multiple determination.

*p < 0.05.

## Discussion

This study demonstrated the alteration in the levels of DA and other monoamine neurotransmitters in peripheral body fluids by quantifying plasma monoamine levels and their urinary metabolites in PD patients. As there have been few reports determining these chemicals in plasma and urine of PD patients, we would discuss by comparing our results to the previous ones in central body fluid, imaging, or post-mortem brain tissue studies instead.

For the dopaminergic system, our analyses showed that PD patients have significantly higher urinary HVA levels and HVA/DA ratio than control subjects. Additionally, in PD patients, the plasma DA level increased in parallel with the higher LEDD. These findings are consistent with a previous study by Andersen et al. (2017) reporting increased DA and HVA levels in the cerebrospinal fluid of PD patients treated with L-DOPA, and a decrease in untreated PD patients. When nigrostriatal degeneration progresses in PD, the surviving dopaminergic neurons compensate the loss by increasing DA synthesis, storage, release, and turnover through upregulation of aromatic amino acid decarboxylase (AADC) and vesicular monoamine transporter type 2 (VMAT2) (Lee et al., 2000). These compensatory responses may explain why DA and HVA were increased in PD patients. The activity of monoamine oxidase (MAO) in PD patients is also increased (Lee et al., 2000). The rise in HVA thus appears to be greater than the rise in DA.

L-DOPA administration may be an additional factor causing abnormal increases in DA and its metabolite. L-DOPA can be taken up by non-dopaminergic neurons, particularly serotonergic and noradrenergic neurons and astrocytes, leading to the increase in production of DA as these neurons possess plentiful AADC and VMAT2 which are essential for DA synthesis and storage (Carta et al., 2008a, b; Pavese et al., 2011). Furthermore, long-term use of L-DOPA also stimulates angiogenesis and changes the permeability of the blood–brain barrier to increase its diffusion into the brain (Ohlin et al., 2011). All the above factors may be implicated in the rise of DA levels in the synaptic cleft, extracellular fluid, and peripheral circulation (Sossi et al., 2007). In this study, we attempted to minimize the effect of L-DOPA by having the patients discontinue their medications for 12 h prior to specimen collection, which is much longer than the half-life of L-DOPA.

Among PD patients, we also found that the plasma DA level decreased in parallel with the longer disease duration. Similarly, Lunardi et al. (2009) reported a negative correlation between DA level in CSF and disease duration in PD patients. Additionally, they found that the HVA/DA ratio was higher in the patients with a longer disease duration (Lunardi et al., 2009). Therefore, the negative relationship between DA level and disease duration may be explained by both the progressive degeneration of dopaminergic neurons and the abnormal increase in DA degradation.

Regarding NE and EPI, this study demonstrated significantly increased plasma NE with a decreased VMA/NE ratio in PD patients, indicating that they have a high rate of NE synthesis with a low rate of its degradation. The plasma EPI level was significantly lower in PD than in control groups, while the VMA/EPI ratio was not different between the groups. Information regarding the alterations of NE and EPI remains controversial. A study by Chia et al. (1993) showed that PD patients had a higher plasma NE without a difference in plasma EPI level compared to control subjects. Similarly, Andersen et al. (2017) revealed that the NE level in CSF was significantly increased, whereas the ratio of methoxy-4-hydroxyphenylglycol (MHPG)/NE was significantly decreased in PD patients treated with L-DOPA. On the other hand, a study by Eldrup revealed no differences in plasma DA, NE, and EPI between PD patients and control subjects (Eldrup et al., 1995). In an electrophysiology study, it was reported that the firing rate of noradrenergic neurons in the locus coeruleus was increased in PD rats compared to normal ones (Wang et al., 2009). Normally, DA is converted into NE and EPI by the catalyzed activities of dopamine beta-hydroxylase (DBH) and phenyl-ethanolamine-N-methyltransferase enzyme (PNMT), respectively. The study by Kopp et al. (1982) suggested that PD patients had enhanced activity of DBH without change in PNMT activity in the brainstem. This may be the reason why PD patients in our study had increased NE, but not EPI, levels.

For the serotonergic system, we found that PD patients had remarkably decreased plasma 5-HT and urinary 5-HIAA. However, the ratio of 5-HIAA/5-HT in PD was not different from the control group. From these results, it can be assumed that PD patients have a reduced 5-HT synthetic rate but unchanged turnover rate. These results agree with several previous studies which reported that 5-HT and 5-HIAA levels were significantly lower in PD patients compared to healthy controls, in both CSF and peripheral circulation (Olivola et al., 2014; Tong et al., 2015). The decrease of 5-HT may result from Lewy body deposition and subsequent destruction of raphe nuclei (Braak et al., 2003). Moreover, many studies suggest that L-DOPA could inhibit 5-HT production. As mentioned earlier, serotonergic neurons are susceptible to uptake of L-DOPA, which might act as a competitive inhibitor of 5-HT synthesis (Goldstein and Frenkel, 1971).

This study also revealed that PD patients in the advanced stage (i.e., high motor severity) had significantly lower plasma 5-HT levels than patients in the early stage (i.e., low motor severity). This association is similar to a previous study regarding 5-HT dysfunction, and positron emission tomography (PET) study, which revealed a greater loss of serotonergic terminals at the raphe nuclei and striatum in advanced PD compared to early PD patients (Politis et al., 2010). Another two PET studies also reported that 5-HT transporter-binding markers and 5-HT_1__*A*_ receptors in the raphe nuclei, caudate, and putamen negatively correlated with tremor severity in PD patients (Doder et al., 2003; Loane et al., 2013). Moreover, Coppen et al. (1972) showed that tryptophan co-treatment in PD patients was more effective in improving motor symptoms. Thus, 5-HT depletion may contribute to the severity of motor impairment in PD.

This study is limited by the small number of participants. In addition, a concurrent study on the activities of enzymes related to monoamine metabolism may lead to a better understanding of the monoamine system changes in PD. Furthermore, the measurement of monoamines and metabolites in peripheral body fluid may not precisely reflect their levels or activities in the central nervous system. However, our findings are in the same direction with several studies measuring monoamine levels in CSF or determining their activities by the neuroimaging technique. Considering this, the potential use of monoamine level measurement in peripheral body fluid as PD biomarkers should be investigated in future studies.

In conclusion, our study demonstrated the alteration of monoamine neurotransmitter in peripheral body fluids of PD patients. Correlations between disease severity and plasma 5-HT level, as well as disease duration and plasma DA level, were also demonstrated. This information contributes to our wider knowledge of multi-neurotransmitter dysfunction in PD, thus enhancing the evaluation of neurotransmitter status, prediction of subsequent symptoms, planning of appropriate disease management, and monitoring of the effectiveness of treatments.

## Data Availability Statement

The original contributions presented in the study are included in the article/supplementary material, further inquiries can be directed to the corresponding author.

## Ethics Statement

The studies involving human participants were reviewed and approved by Internal Review Board, Faculty of Medicine, Chulalongkorn University. The patients/participants provided their written informed consent to participate in this study.

## Author Contributions

PW acquired the data and drafted the manuscript. PW, ST, and SB-p analyzed and interpreted the data. ST, SB-p, RB, and OP revised the manuscript. ST and SB-p obtained the funds. All authors designed the study and approved the final version of the manuscript.

## Conflict of Interest

The authors declare that the research was conducted in the absence of any commercial or financial relationships that could be construed as a potential conflict of interest.

## Publisher’s Note

All claims expressed in this article are solely those of the authors and do not necessarily represent those of their affiliated organizations, or those of the publisher, the editors and the reviewers. Any product that may be evaluated in this article, or claim that may be made by its manufacturer, is not guaranteed or endorsed by the publisher.

## Footnotes

1
www.chulapd.org
